# Wharton’s jelly‐derived stromal cells and their cell therapy applications in allogeneic haematopoietic stem cell transplantation

**DOI:** 10.1111/jcmm.17105

**Published:** 2022-01-28

**Authors:** Cécile Pochon, Anne‐Béatrice Notarantonio, Caroline Laroye, Loic Reppel, Danièle Bensoussan, Allan Bertrand, Marie‐Thérèse Rubio, Maud D’Aveni

**Affiliations:** ^1^ Pediatric Oncohematology Department CHRU Nancy Université de Lorraine Nancy France; ^2^ UMR 7365 CNRS IMoPA Université de Lorraine Nancy France; ^3^ Hematology Department CHRU Nancy Université de Lorraine Nancy France; ^4^ Cell Therapy Unit CHRU Nancy Université de Lorraine Nancy France

**Keywords:** applications, cell therapy, graft versus host disease, mesenchymal stem cells, stem cell transplantation, stromal cells, Wharton's jelly

## Abstract

For decades, mesenchymal stromal cells (MSCs) have been of great interest in the fields of regenerative medicine, tissue engineering and immunomodulation. Their tremendous potential makes it desirable to cryopreserve and bank MSCs to increase their accessibility and availability. Postnatally derived MSCs seem to be of particular interest because they are harvested after delivery without ethical controversy, they have the capacity to expand at a higher rate than adult‐derived MSCs, in which expansion decreases with ageing, and they have demonstrated immunological and haematological supportive properties similar to those of adult‐derived MSCs. In this review, we focus on MSCs obtained from Wharton's jelly (the mucous connective tissue of the umbilical cord between the amniotic epithelium and the umbilical vessels). Wharton's jelly MSCs (WJ‐MSCs) are a good candidate for cellular therapy in haematology, with accumulating data supporting their potential to sustain haematopoietic stem cell engraftment and to modulate alloreactivity such as Graft *Versus* Host Disease (GVHD). We first present an overview of their in‐vitro properties and the results of preclinical murine models confirming the suitability of WJ‐MSCs for cellular therapy in haematology. Next, we focus on clinical trials and discuss tolerance, efficacy and infusion protocols reported in haematology for GVHD and engraftment.

## 1 INTRODUCTION

Mesenchymal stromal cells (MSCs) were first described in bone marrow and defined as non‐haematopoietic, fibroblast‐like, plastic‐adherent cells.^1^ Since their first discovery, they have been described in other adult tissues^2^ and in foetal/perinatal tissues.^3^ Though derived from different tissues, they do share some common features^4^: plastic adherence; the expression of stromal surface markers such as CD73, CD90 and CD105, and no expression of haematopoietic CD34, CD45, CD14/CD11b, CD79α/CD19 or MHC class II (human leukocyte antigen HLA‐DR); and the capacity to differentiate into several mesenchymal cell types including osteoblasts, chondroblasts and adipocyte progenitors.

MSCs have been isolated from multiple adult tissues other than bone marrow, including skeletal muscle, adipose tissue, synovial membranes, saphenous veins, dental pulp, periodontal ligaments, lung, liver and skin. MSCs obtained from extra‐embryonic tissues (such as placenta/umbilical cord, Wharton's jelly and amniotic membrane) share properties with their adult counterparts. They also retain characteristics of primitive stem cells, like the expression of the embryonic stem cell markers Oct‐4, Nanog, Sox‐2 and c‐Kit, as well as Dnmt3b and hTERT, albeit at much lower levels than those of embryonic stem cells.^5^ They have many advantages for cellular therapy applications: available after delivery, their collection and expansion raise no ethical issues. They usually expand with a higher proliferation rate^6^ and a broader multipotency than MSCs from adult tissues, though donor age affects these characteristics in bone marrow‐derived MSCs (BM‐MSCs).^7^

Wharton's jelly mesenchymal stromal cells (WJ‐MSCs), were first isolated in 1991.^8^ Their immunomodulatory properties and their low cellular immunogenicity^9^ make them remarkably interesting for the induction of tolerance in transplantation. A few in vitro studies have reported that WJ‐MSCs are less immunogenic than MSCs from other sources, especially when cultivated in hypoxia.^10^ Amongst MSCs, WJ‐MSCs have the lowest level of MHC class II expression. Moreover, they express low levels of cell‐surface MHC class I and costimulatory molecules (CD40, CD80, CD86). In a comparative study of foetal adnexa‐derived caprine MSCs, WJ‐MCs outperformed MSCs from other sources of foetal adnexa (amniotic fluid, amniotic sac, cord blood) in terms of growth kinetics, relative messenger ribonucleic acid (mRNA) expression of surface antigens, pluripotency markers, and tri‐lineage differentiation potential.^11^

In allogeneic haematopoietic stem cell transplantation (allo‐HSCT), BM‐MSCs were the first described cells of potential interest. They have been mainly (and widely) studied in the treatment of steroid‐refractory graft versus host disease (GVHD), with results varying depending on the study and the methods used.^12^, ^13^, ^14^ Another topic of research has been their haematopoietic support functions since they are key players in the haematopoietic niche and can enhance haematopoietic stem cell (HSC) engraftment and function.^15^, ^16^, ^17^ However, in this area of research, their efficacy remains to be established.^15^, ^18^

Several studies, which we describe below, have compared MSCs isolated from the most common sources in terms of their functional differences in immune modulation and haematopoietic support.

### 1.1 Immunosuppression

MSCs derived from amnion, placenta, Wharton's jelly and umbilical cord blood elicit a similar degree of immunosuppression in vitro compared with that of BM‐MSCs, but Manochantr et al. have demonstrated that WJ‐MSCs have a higher proliferative capacity.^19^ MSCs derived from WJ and adipose tissue have potent immunosuppressive effects on T cells, similar^20^ or even superior to that observed with BM‐MSCs.^21^ WJ‐MSCs have a capacity to suppress neutrophil adhesion to inflamed endothelium similar to that of BM‐MSCs, but conserve their immune‐suppressive properties after passage 7, unlike BM‐MSCs.^22^ In a murine model of experimental sepsis, mice treated with WJ‐MSCs had a higher survival rate than mice treated with BM‐MSCs.^23^ In another study, by Karaöz et al, WJ‐MSC co‐culture with activated T cells led to a higher concentration of IL‐17A, which would be of particular interest in a GVHD context.^24^ In a recent work, Shin et al. analysed the protein secretome of four sources of MSCs: adipose tissue, bone marrow, placenta, and WJ. Each MSC secretome profile had distinct characteristics, depending on the source. The secretome of foetal‐derived MSCs (placenta and WJ) had a more diverse composition than those of adipose tissue and BM‐MSCs, and the authors assumed that their therapeutic potential was greater because of these properties.^25^

### 1.2 Haematopoiesis

One study found more proteins related to tissue development and the differentiation of haematopoietic cells in the secretome of WJ‐MSCs compared with adipose tissue and BM‐MSCs.^25^ Moreover, CD117 (c‐kit) the receptor for stem cell factor (SCF) harboured by haematopoietic stem/progenitor cells (HSPCs), has been repeatedly detected in WJ‐MSCs.^26^ WJ‐MSCs express osteopontin and are able to secrete hyaluronic acid.^27^ Interestingly, both these molecules are amongst the main constituents of the HSPC niche. Osteopontin is a critical regulator of HSPC localization and proliferation.^27^, ^28^ Human WJ‐MSCs have been compared with BM‐MSCs and show similar haematopoiesis‐supportive functions in vitro, when co‐cultured with CD34^+^ umbilical cord blood cells.^29^ In a murine model, co‐transplantation of either WJ‐MSCs or BM‐MSCs with CD34^+^ HSCPs from cord blood resulted in similarly enhanced recoveries of human platelets and CD45^+^ cells in the peripheral blood and a 3‐fold higher engraftment in the bone marrow, blood, and spleen 6 weeks after transplantation when compared with transplantation of CD34^+^ cells alone.^30^

**FIGURE 1:**
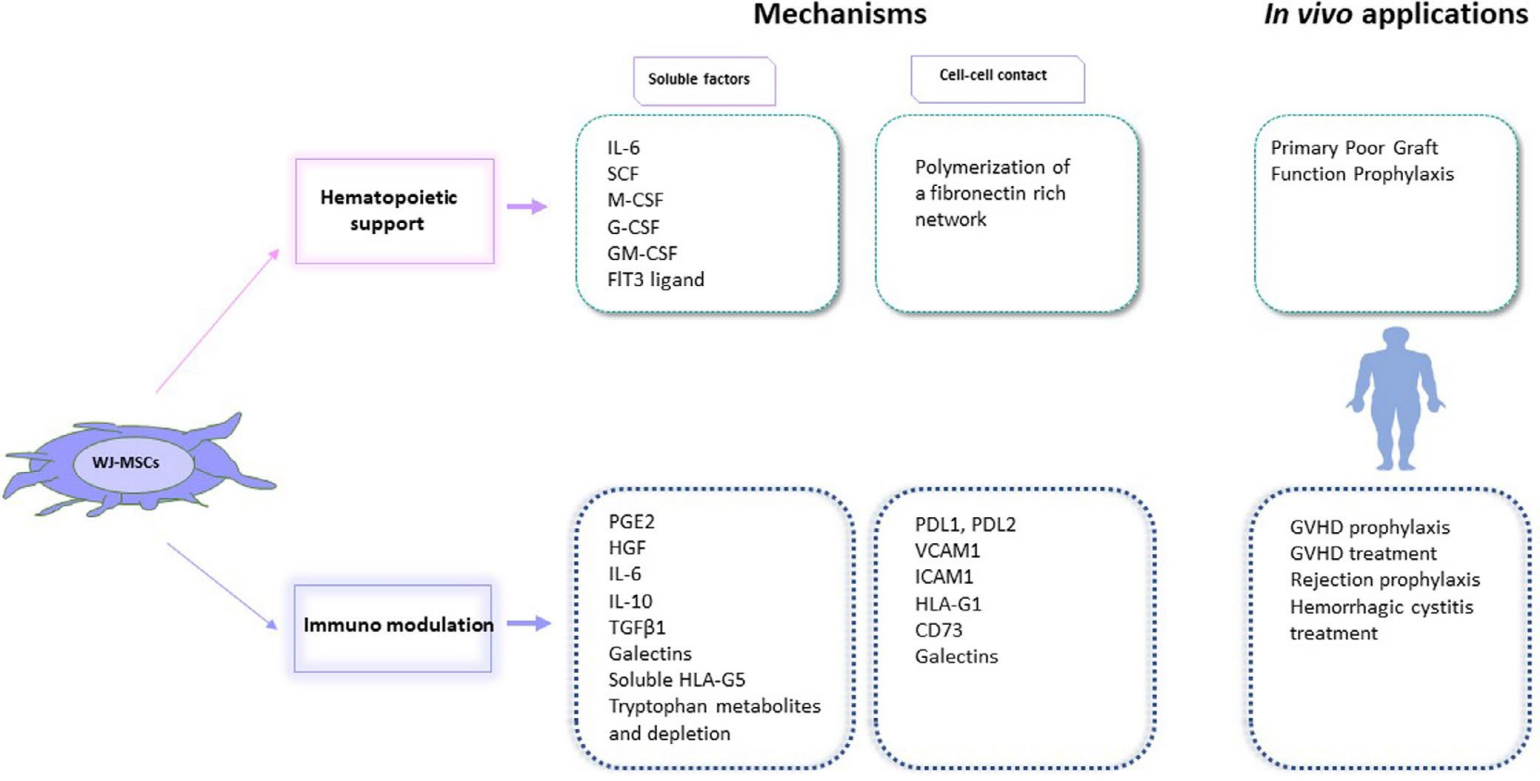
WJ‐MSCs support haematopoiesis and modulate immunity via soluble factors and cell‐cell contact. *Upper*: WJ‐MSCs secrete growth factors that may enhance haematopoietic cell renewal or stemness, and they may create a fibronectin network that supports haematopoietic cell homeostasis. Thus, they are of interest in the treatment of poor graft function after HSCT. IL‐6: Interleukin‐6, SCF: stem‐cell factor, M‐CSF: macrophage colony‐stimulating factor, G‐CSF: granulocyte colony‐stimulating factor, GM‐CSF: granulocyte macrophage colony‐stimulating factor, Flt3: Fms‐like tyrosine kinase 3; *Lower*: WJ‐MSCs secrete cytokines and other molecules that decrease activated T‐cell proliferation, or induce Tregs, and act on other immune cells. They also produce cytosolic IDO, an enzyme that depletes tryptophan in the medium and converts tryptophan into secreted metabolites (like kynurenine) that prevent T‐cell proliferation. WJ‐MSCs also express several membrane molecules that interact with activated T cells to induce exhaustion or apoptosis, or to prevent T‐cell activation. The expression of soluble and membrane factors varies according to the level of inflammation in the environment. These properties make WJ‐MSCs good candidates for GVHD prophylaxis or cure, for graft rejection prophylaxis, and for some disorders of uncontrolled inflammation, such as haemorrhagic cystitis. PGE2, prostaglandin E2; HGF, hepatic growth factor; IL, interleukin; TGF β1, transforming growth factor β1; HLA, human leukocyte antigen; PDL (1/2), programmed‐death ligand; VCAM, vascular cell adhesion molecule; ICAM, intercellular adhesion molecule

Considering their low immunogenicity, their immunomodulatory and haematopoietic supportive properties in vitro, their growth kinetics, and their proteome diversity, WJ‐MSCs are remarkably interesting cells for potential use in GVHD and haematopoietic engraftment (Figure 1).

## 2 WJ‐MSCS SUSTAIN HAEMATOPOIESIS

In the setting of sustaining haematopoiesis, MSCs from placenta and umbilical cord display interesting properties in vitro.^30^ They promote growth and preserve the stemness of haematopoietic stem cells (HSCs) from autologous or allogeneic cord blood in two‐dimensional cultures,^31^, ^32^ and in three‐dimensional scaffolds.^33^ WJ‐MSCs have been recently exploited as a feeder layer to expand haematopoietic stem cells, providing secreted proteins^34^ and cytokines involved in the regulation of haematopoiesis, including interleukin (IL)‐6, SCF, Fms‐like tyrosine kinase 3–ligand (Flt‐3L), macrophage colony‐stimulating factor, granulocyte colony‐stimulating factor and granulocyte‐monocyte colony‐stimulating factor (Table 1).^35^

**TABLE 1:** WJ‐MSC infusions and clinical applications in haematology (cord blood MSCs excluded)

Indications	Authors Country	Number of patients/ Phase Trial	Infusion dose	Schedule	Results	Side effects
GVHD Treatment	Soder et al^50^ USA	N = 10, adults Phase 1	2 or 10 × 10^6^ MSCs/kg/infusion	2 infusions: on day 0 and 7 of aGVHD treatment	ORR 70%, CR 40%, PR 30%	No acute infusion‐related toxicity No treatment‐related adverse events (TRAE)^a^ No ectopic tissue formation
	Wu et al^54^ China	N = 24 phase 2	0.6 × 10^6^/kg Range (0.5–1.0) ×10^6^/kg	Single infusion Refractory aGVHD and cGVHD	55.6% of improvements for skin, 100% for oral mucosa, 37.5% for GI tract, no response for liver and lung	No acute infusion‐related toxicity No TRAE^a^
GVHD Prophylaxis	Zhu et al^51^ China	N = 25, children Phase 1–2	Mean = 1.14 × 10^6^ MSCs/kg Range (1.03–1.39) ×10^6^/kg	Single infusion 1 hour before HSCT	2 patients severe late onset aGVHD 2 patients extensive cGVHD	No acute infusion‐related toxicity Adverse events:CMV infection in 23 patients, bacterial and fungal infections, treatment related?
	Wu et al^52^ China	N = 50 Phase 2	Mean = 5 × 10^5^ MSCs/kg	Single infusion 4 hours before HSCT	12/50 aGVHD gr II‐IV 3 extensive cGVHD	No acute infusion‐related toxicity TRAE^a^ observed in 9 patients
	Gao et al^53^ China	N = 124 Multicentre, double‐blind, randomized Phase 2	Mean = 3 × 10^7^/100 ml/month Range?	Repeated infusions 1/month ×4 >4 months after HSCT	3/62 severe cGVHD *versus* 8/62 in the control group cGVHD = 27.4% in the MSCs group *versus* 49% in the control group (*p* = 0.021)	No acute infusion‐related toxicity No increase in adverse events^a^: infections and grade 1–2 liver dysfunction and renal impairment observed in 45 patients (72.6%) in the MSCs group and 40 patients (64.5%) in the non‐MSC group
Engraftment and GVHD	Wang et al^37^ China	N = 22, SAA Phase 1–2	Mean = 1.2 × 10^6^ MSCs/kg Range (0.27–2.5) ×10^6^/kg	Single dose 4 hours before HSCT	22 engraftments, no severe aGVHD nor cGVHD Long‐term full donor chimerism for 21/22 patients	Two patients had slight fever immediately after cell injection, which resolved within 12 hours. No TRAE^a^
	Li et al^38^ China	N = 17, SAA adults Phase 1–2	Mean = 4 × 10^6^ MSCs/kg Range (2.87–10) ×10^6^/kg	Single infusion 6 hours before HSCT	16/17 engraftments with full donor chimerism, 1/17 graft failure 4/17 grade III‐IV aGVHD 6/17 cGVHD (1 severe)	No acute infusion‐related toxicity Adverse events: CMV and/or EBV infections in 10 patients. Treatment related?
	Wang et al^39^ China	N = 17, SAA Children Phase 1–2	Median dose 4 × 10^7^ (0.5–8)	Single infusion On day +1 after HSCT	17/17 myeloid engraftments 16/17 platelet engraftments 1 grade III‐IV aGVHD cGVHD = 21.2% 1 secondary graft failure OS = 71%	No acute infusion‐related toxicity Adverse events were not related to MSC infusion: Three patients died of TRM, because of infection on day +36, severe aGVHD on day +44 and viral interstitial pneumonia on day +634
	Wu et al^36^ China	N = 20 (8 patients in the MSC group) Cord blood transplantation Randomized phase 2	Median dose of 7.19 × 10^6^ MSCs/kg (2.44–10.12)	Single infusion 4 hours before Cord blood	Faster recovery of ANC (*p* = 0.003) and platelets (*p* = 0.004) in the MSC group	No acute infusion‐related toxicity No ectopic tissue on MRI and PET survey in the interval of 3 months after WJ‐MSCs infusion. No CMV disease, septic shock, nor fungal infection. TRAE^a^ observed in 5 patients (4 mild GVHD and 1 relapse). No deaths related to treatment toxicity. (2 deaths related to infections, and 2 relapses in the comparative group)
	Wu et al^40^ China	N = 21, SAA Phase 1–2	5 × 10^5^ MSCs/kg Invariable dose	Single infusion 4 hours before HSCT	21/21 sustained engraftments 2‐y PFS = 74% 5 grade III–IV aGVHD 3 extensive cGVHD	No acute infusion‐related toxicity TRAE^a^ observed in 4 patients

Note

Abbreviations: cGVHD, chronic GVHD); CMV, cytomegalovirus; CR, complete response; EBV, Epstein‐Barr virus; GI, gastrointestinal; GVHD, graft *versus* host disease (aGVHD, acute GVHD; ORR, overall response; PFS, progression‐free survival.; PR, partial response; SAA, severe aplastic anaemia.

^a^ Treatment‐related adverse events (TRAE) were graded using the National Cancer Institute Common Toxicity Criteria for Adverse Events (CTCAE version 4.0) and commonly include oral ulcer, diarrhoea, gastrointestinal haemorrhage, haemorrhagic cystitis, interstitial pneumonia, and liver or renal dysfunction, and, rarely, relapse and GVHD.

### 2.1 Preclinical studies

Studies in NOD/SCID/IL2Rγnull (NSG) mice suggest that WJ‐MSCs may increase haematologic recovery.^36^ Six weeks after allogeneic stem cell transplantation, NSG mice co‐transplanted with 1 × 10^6^ WJ‐MSCs demonstrated a significantly higher median number of human CD45^+^ cells engrafting in the peripheral blood and bone marrow than those transplanted without WJ‐MSCs: respectively, 28.2% (range, 24.6–33.1%) versus 5.3% (range, 4.2–6.5%) and 6.9% (range, 5.9–7.3%) versus 1.7% (range, 1.5–2.3%).

### 2.2 Clinical studies

Severe aplastic anaemia (SAA) is a life‐threatening bone marrow failure. Allo‐HSCT with alternative donors (unrelated or mismatched donors, cord blood) is usually reserved for SAA patients who do not have a matched sibling and who have not responded to immunosuppressive drug therapy first, because of the poor outcome after allo‐HSCT in this context (high risk of rejection, poor graft function with haemorrhagic and infectious complications, and high GVHD incidence). The first report in SAA of co‐injection of WJ‐MSCs and allo‐HSCT for these patients who have not responded to immunosuppressant was based on 22 patients.^37^ Eight patients received haploidentical haematopoietic stem cells, six patients had matched related donors, and eight patients had matched unrelated donors. WJ‐MSCs were obtained from umbilical cords as follows: the main vessels were removed and the jelly was digested using 1 mg/ml collagenase. Cell culture was normoxic, and cells were cryopreserved before use. WJ‐MSCs were selected if they matched at least three HLA alleles with recipients, then expanded and cryopreserved a second time. They were infused into patients immediately after thawing, at an average dose of 1.2 × 10^6^/ kg (range, 0.27–2.5 × 10^6^/kg). None of the patients experienced graft rejection, and all had rapid engraftment (mean times for neutrophil and platelet recovery were 13.95 days and 20.27 days, respectively). No severe acute GVHD (aGVHD) and no chronic GVHD (cGVHD) were observed. With a median follow‐up of 15 months, 21 patients were alive and transfusion‐independent with full donor chimerism. This report has been confirmed in 17 SAA adult patients treated with haploidentical HSCT after a reduced‐intensity conditioning regimen and with co‐infusion of culture‐expanded third‐party donor‐derived WJ‐MSCs 4 hours before allo‐HSCT.^38^ In this study, WJ‐MSCs were obtained after the cord was sectioned, without enzymatic digestion. Cells were thawed only once, in autologous plasma from cord blood, and re‐expanded before their use at day 0 of HSC transplantation. Moreover, 17 children and adolescents with SAA were treated with haploidentical HSCT after myeloablative conditioning and co‐infusion of culture‐expanded third‐party donor‐derived WJ‐MSCs (obtained from cords after vessel removal and enzymatic digestion, and used fresh at passage 3) at day +1 post transplantation and they experienced the same good outcomes.^39^ In these two studies, 16/17 and 17/17 patients, respectively, achieved complete haematopoietic reconstitution. Median times to neutrophil count >0.5 × 10^9^/L were 12 and 16 days, respectively, and to platelet count >20 × 10^9^/L were 14 and 22 days, respectively. These promising preliminary data paved the way for other studies, with encouraging results.^40^ A randomized phase 2 study demonstrated that a WJ‐MSC (1 × 10^6^ WJ‐MSC/kg) infusion 4 hours before transplantation is correlated with a faster haematologic recovery of neutrophils and platelets in UCB transplantation.^36^ WJ‐MSCs were isolated once again after vessel removal from the cord, enzymatic digestion, normoxic culture with foetal bovine serum and two cryopreservations. Taken together, these data suggest that WJ‐MSCs improve haematologic reconstitution by shortening the median time to neutrophil and platelet recovery. However, meta‐analyses are necessary to better assess the effect of WJ‐MSCs on HSC engraftment. In a recent meta‐analysis of BM‐MSC and WJ‐MSC infusions for SAA patients in haploidentical HSCT, no improvement on overall survival nor on toxicity‐related mortality was proven when MSCs were co‐infused with HSCs.^18^

## 3 WJ‐MSCS AS A PROMISING THERAPY TO MODULATE GVHD

### 3.1 Preclinical studies

**FIGURE 2:**
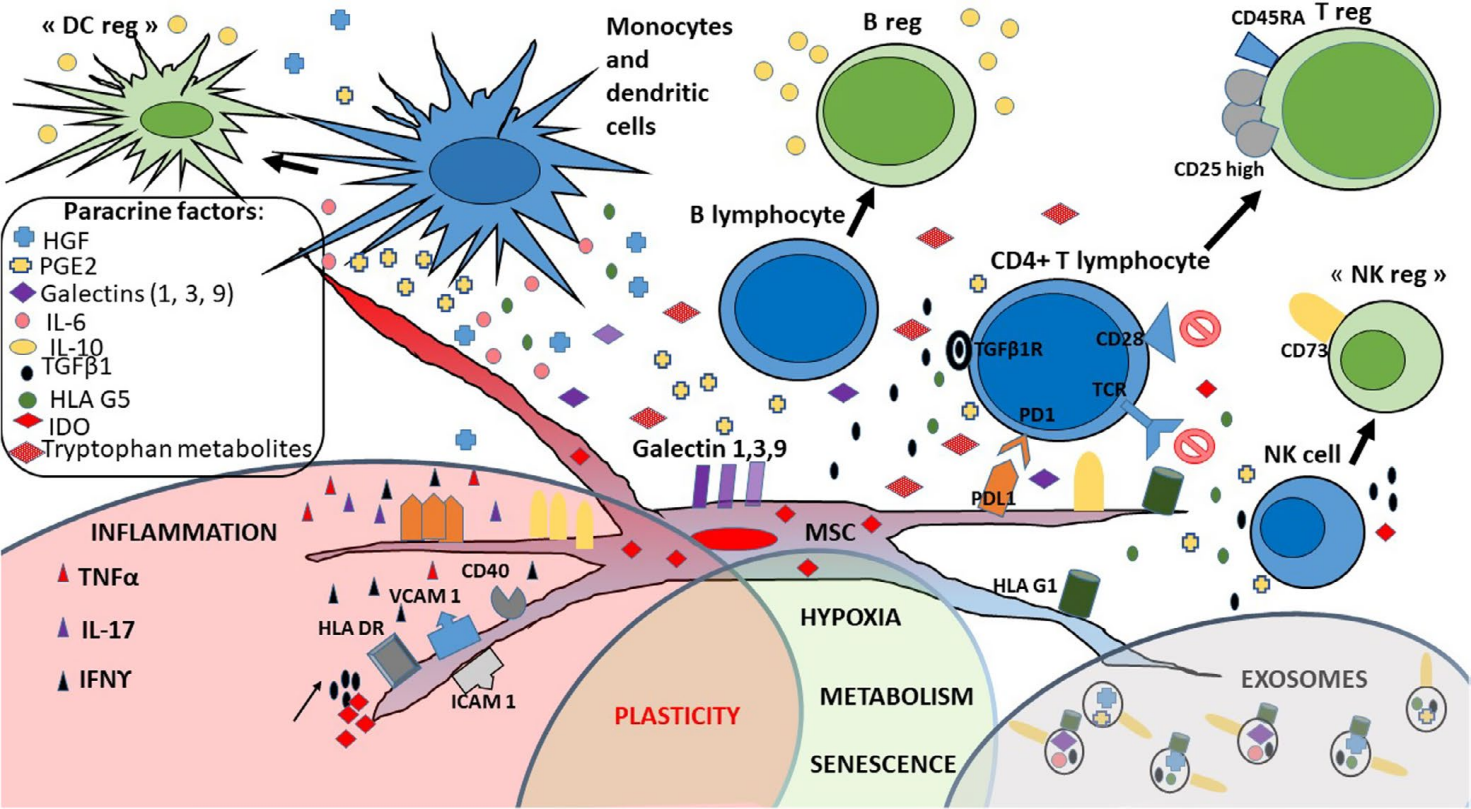
WJ‐MSCs use several membrane and soluble factors to modulate the immune system. WJ‐MSCs regulate immunity through cell‐cell contact with T cells: PD‐L1 (programmed‐death ligand 1), galectins (1, 3, 9), VCAM 1 (vascular cell adhesion molecule 1), ICAM 1 (intercellular adhesion molecule 1), HLA (human leukocyte antigen)‐G1, and CD73. They also express the usual HLA class I molecules and do not express costimulatory molecules. They may express CD40 and HLA class II molecules in an inflamed environment. They produce many soluble factors: PGE2 (prostaglandin E2), HGF (hepatocyte growth factor), IL (interleukins) −6 and −10, TGFβ1 (transforming growth factor β1), soluble HLA‐G5, and soluble galectins (1, 3 and 9). These soluble factors decrease T‐cell proliferation, induce T‐cell apoptosis and polarize T cells to become regulatory T cells (Tregs). They also prevent dendritic cell (DC) maturation and modify NK and B‐cell functions, giving them a ‘regulatory’ phenotype. Moreover, WJ‐MSCs express IDO (indoleamine 2–3 dioxygenase), in their cytosol. This enzyme is responsible of tryptophan depletion in the medium, and tryptophan‐metabolite production (kynurenine, 3‐hydroxykynurenine and kynurenic acid)

Whilst immunomodulatory effects of BM‐MSCs mainly rely on cell‐to‐cell contact, soluble factors with immunomodulatory effects are more frequently described for WJ‐MSCs. Indoleamine 2,3‐dioxygenase 1 (IDO) has been identified as one of the main effector molecules responsible for T‐cell suppression in MSCs from all sources.^41^ WJ‐MSCs also produce regulatory cytokines such as transforming growth factor‐β1 (TGF‐β1), regulatory soluble factors such as vascular endothelial growth factor (VEGF); hepatocyte growth factor (HGF); galectins 1, 3, and 9; prostaglandin E2 (PGE2); soluble HLA‐G5 or membrane HLA‐G; and programmed‐death ligands 1 and 2 (PD‐L1 and PD‐L2). Moreover, amongst MSCs, they express the highest levels of the adhesion markers CD29 (β1), CD49d (α4), and CD54 (ICAM‐1),^42^, ^43^ and produce higher amounts of IL‐1Rα, RANTES, IP‐10, and MCP1 chemokines.^44^ This might impact their homing to the inflamed tissues and their fate after injection. Indeed, it has been shown that they can be quickly phagocytosed by monocytes after their injection into mice, and that phagocytosis induces changes in monocytes (CD14^++^, CD16^+^, CD206^+^, PD‐L1^+^, IL‐10^+^), which subsequently modulate adaptive immune cells.^45^ Differences observed between these various immunomodulatory mechanisms might be due to differences in WJ‐MSC plasticity depending on the isolation procedure, culture techniques, the inflammatory context of a chosen preclinical model, and variation amongst donors according to obstetrical factors^46^, ^47^ (Table 1, Figure 2).

Since the mid‐2000s, cultured/expanded WJ‐MSCs have been tested for their therapeutic potential in preclinical animal models of allo‐HSCT. Several studies suggest that WJ‐MSCs may prevent graft‐versus‐host disease (GVHD) especially with repeated injections.^48^ Before GVHD onset, 5 × 10^5^ WJ‐MSCs /mouse significantly improved the survival rate only when they were repeatedly injected at 3‐day intervals. In contrast, a single injection of WJ‐MSCs after GVHD onset is enough to significantly increase the survival rate and effectively attenuate tissue damage. Finally, in preclinical studies WJ‐MSCs have also been reported to decrease the cumulative incidence and the severity of GVHD^49^: 1 × 10^6^ WJ‐MSCs per mouse increased the survival rate when injected two times at 7‐day intervals. This result was similar to that achieved after only one infusion of WJ‐MSCs, primed by IFN‐γ, 24 hours prior to injection.

### 3.2 Clinical studies

#### 3.2.1 GVHD treatment

The first phase 1 trial published with WJ‐MSC administration in the treatment of acute GVHD was performed in the USA. WJ‐MSCs were used immediately after thawing but the culture conditions were not described. Ten patients with de novo high risk or steroid‐refractory acute GVHD received WJ‐MSCs intravenously on days 0 and 7 (low‐dose cohort, 2 × 10^6^/kg, n = 5; high‐dose cohort, 10 × 10^6^/kg, n = 5). No infusion‐related toxicity, treatment‐related adverse events, or ectopic tissue formation was observed in either cohort. Clinical response was suggested at day 28, as the overall response rate (ORR) was 70%, with a complete response in 4 out of 10 patients, and a partial response in 3 patients. REG3α (a serum biomarker of acute GVHD) decreased, particularly in the high‐dose cohort, and this was correlated with clinical response. Overall survival rates at day 100 and day 180 post infusion were 90% and 60%, respectively.^50^ This publication represents an important step forward in the treatment of acute severe steroid‐refractory GVHD, with repeated injections of high‐dose WJ‐MSCs (10 × 10^6^ cells/kg) that should be tested in expanded, randomized and controlled trials. Currently, repeated infusions at a dose of 1–3 × 10^6^/kg/week of WJ‐MSC in the treatment of steroid‐resistant severe acute GVHD are being explored for safety and efficacy in Europe (NCT01092026 in Belgium and NCT02032446 in Italy).

#### 3.2.2 GVHD prophylaxis

In the setting of prophylactic administration of WJ‐MSCs against GVHD, three studies have reported the results of co‐infusion with WJ‐MSCs at the time of haploidentical transplantation for malignant haematological diseases. Unfortunately, none of these studies was performed with a control group. In the first study,^51^ 25 children 4–17 years old were enrolled: 7 patients were diagnosed with acute myeloid leukaemia (AML), 17 with acute lymphoblastic leukaemia (ALL), and one with bi‐phenotypic acute leukaemia, and all were considered high risk before transplant. WJ‐MSCs were given by intravenous infusion at a mean dose of 1.14 × 10^6^/kg (range: 1.03–1.39 × 10^6^/kg) over 30 minutes, followed by an infusion of haploidentical haematopoietic cells. WJ‐MSCs were isolated after cord vessel removal, enzymatic digestion, culture in a medium without animal or human proteins, and used fresh before passage 5. All patients had a rapid myeloid engraftment with complete donor chimerism at 1‐month post‐transplant. Only three patients experienced a stage II acute GVHD, two patients presented severe late onset acute GVHD and two patients presented extensive chronic GVHD. Of note, this study highlighted a concern about high incidences of infection and relapse. Cytomegalovirus (CMV) replication was observed in 23 patients and successfully treated with ganciclovir. Eleven patients died, including six because of relapse. These high incidences of infection and relapse were not confirmed by the other studies. In the second study, 50 patients aged from 9 to 58 years old with haematologic malignancy were enrolled. Patients (23 acute myeloid leukaemia and 17 acute lymphoid leukaemia, 7 lymphomas and 3 chronic myeloid leukaemia's in blast crisis) were transplanted with haploidentical and mismatched‐unrelated donors. They received a busulfan‐based myeloablative conditioning regimen. Co‐infusion at a lower dose of WJ‐MSCs (5 × 10^5^/kg) was performed 4 hours prior to allo‐HSCT. WJ‐MSCs were isolated without enzymatic digestion and thawed in autologous plasma (from cord blood). They were infused immediately after thawing. All 50 patients achieved full donor chimerism. Twelve amongst 50 patients (24%) developed a grade II–IV aGVHD and 17 of the 45 (38%) live patients experienced cGVHD, with only 3 cases of extensive cGVHD. Five patients relapsed.^52^ The third study was a multicentre, double‐blind, randomized controlled trial with a delay of more than 4 months following transplantation and with repeated infusions (at 3 monthly infusions on average) of WJ‐MSCs (at a dose of 3 × 10^7^/per month) for cGVHD prophylaxis.^53^ WJ‐MSCs were obtained from the Stem Cell Bank of the Chinese Academy of Sciences and used thawed, but there are no data on cell culture in the article. A total of 124 patients were randomly assigned (WJ‐MSCs, n = 62; control group, n = 62). Seventeen patients (27.4%) had cGVHD, of whom 14 (22.6%) exhibited mild/moderate cGVHD when cyclosporine administration was gradually reduced at the scheduled time, and 3 (4.8%) showed severe cGVHD. In the non‐WJ‐MSC control group, cGVHD occurred in 30 patients (48.4%), of whom 22 (35.5%) exhibited mild/moderate cGVHD, and 8 (12.9%) had severe cGVHD. The cumulative incidence of cGVHD in the WJ‐MSC group was 27.4% (95% CI, 16.2% to 38.6%), compared with 49.0% (95% CI, 36.5% to 61.5%) in the non‐WJ‐MSC control group (*p* = 0.021). Taken together, these studies demonstrate that a single injection prior to allo‐HSCT, at a dose of 5 × 10^5^/kg WJ‐MSCs, seems to be very safe, with no data about efficacy, and a single injection of WJ‐MSCs prior to allo‐HSCT at a dose of 1 × 10^6^/kg may be used with caution. Delayed doses of WJ‐MSC to prevent cGVHD are reported to be a safe and efficient cell therapy. No data are available about a scheme of prophylactic injections administered early after allo‐HSCT with post‐transplantation cyclophosphamide (PTCy).

## 4 WJ‐MSCS MODULATE IMMUNE RECONSTITUTION AFTER TRANSPLANTATION

Only a few studies have sought to determine the impact of WJ‐MSC co‐injection on immune reconstitution during the few months following allo‐HSCT.^53^ In a controlled, randomized multicentre study, four monthly injections of 3 × 10^7^ WJ‐MSCs after haploidentical HSCT for chronic GVHD prevention led to a decrease in total NK cells. Although the numbers of CD3^+^CD4^+^ cells did not differ significantly between the two groups, the number of CD4^+^CD25^+^CD127^low^ regulatory T (Treg) cells in the MSC group was higher than that in the non‐MSC group. And although the numbers of CD19^+^ B cells were not significantly different, the CD27^+^ memory B‐lymphocyte numbers were significantly increased after MSC infusion. Moreover, during the first month post HSCT, the Th1 (interferon γ): Th2 interleukin (IL)‐4^+^ cell ratio increased.^53^ Another study of WJ‐MSC injections in 24 patients with refractory GVHD treatment showed that patients had lower levels of mature dendritic cells (CD83^+^, CD86^+^, and HLA‐DR^+^ cells) after MSC infusion (between days +14 and days +56) than before MSC infusion^54^ (Table 1).

## 5 DISCUSSION

In this review we have presented the reasons why WJ‐MSCs are attracting interest in haematology (summarized in Table 1). They have been described as useful in preventing and treating GVHD, in accelerating HSC engraftment, in SAA patients at high risk of graft failure and poor haematopoietic graft function, and in transplantation procedures (haploidentical HSCT) associated with high morbidity due to GVHD. WJ‐MSCs have been used mostly by Chinese teams (compared with BM‐ and adipose‐derived MSCs in Western countries), but they are gaining interest in Europe and the USA, in haematology and other research areas (eg acute respiratory distress caused by COVID‐19).

Whilst clinical trials are numerous in the setting of allo‐HSCT, there are some barriers to clinical translation.

First, co‐infusion of WJ‐MSCs with allo‐HSCT to prevent GVHD, or the administration of WJ‐MSCs to cure GVHD, encompass various dosages and variable administration schedules of WJ‐MSCs. As prophylaxis for GVHD, WJ‐MSC dosage varies from 2 × 10^5^ to 8 × 10^7^/kg in one infusion (4 hours before transplant) or even in 4 delayed infusions. To treat acute GVHD, WJ‐MSC dosage can vary from 3 × 10^6^/ kg to 10 × 10^6^ WJ‐MSC/ kg in 1 to 3 infusions with an interval of 7 days between each, probably with better results at higher dosages. It is not clear whether, at an equivalent total dose, a single injection or repeated injections can provide similar efficacy. A meta‐analysis found that the minimum effective dose per patient and per trial, regardless of clinical indication, never exceeded 190 × 10^6^ MSCs per injection and that efficacy was never reported at doses lower than 70 × 10^6^ MSCs per injection .^55^ As mentioned above, WJ‐MSCs outperform MSCs from other sources in terms of growth kinetics and maintain their immune‐suppressive functions after a high number of passages, compared with BM‐MSCs. Consequently, administering high numbers of repeated WJ‐MSC injections to patients to ensure the cells’ therapeutic activity is feasible, even from only one cord source. Moreover, there are no data concerning potential allo‐immunization against WJ‐MSCs after repeated injections. Further studies are warranted to better design the optimal scheme of treatment, particularly the timing, the modalities (eg intravenous, intramedullary, etc.), and the number and frequency of injections.

Second, WJ‐MSC injections seem to be a promising means of sustaining haematologic reconstitution after allo‐HSCT, though by an unknown mechanism. In SAA, WJ‐MSCs may well enhance HSC proliferation by modulating autoreactive T lymphocytes. WJ‐MSCs provide a good feeder layer on which to expand HSCs in the very artificial condition of in vitro culture where HSCs are close to WJ‐MSCs. The only previous work demonstrating a significantly higher median number of human CD45^+^ cells in NSG peripheral blood and bone marrow^36^ was performed in a patient‐derived xenotransplantation (PDX) model known for human lymphoid expansion. No preclinical study has compared CD34^+^ engraftment with co‐injection of WJ‐MSCs in specific PDX models developed for myeloid engraftment (eg in NSG‐S or MISTGR mice).

The fate of WJ‐MSCs after intravenous infusion is not well known. To date, no MSCs (whatever their sources) are detectable long term following IV injection in mice. We lack preclinical models and clinical imaging systems to determine where WJ‐MSCs migrate after intravenous infusion and how long they persist in vivo. Only in a murine diabetes model and in Wistar rats do we know that umbilical cord (UC)‐MSCs migrate into the lungs, liver, and spleen.^56^ ^51^Cr‐labelled UC‐MSCs are found in lungs 2 hours after intravenous injection, followed by an accumulation in the liver and spleen from 24 to 96 hours following injection, with hepatobiliary and renal clearance^57^ and a probably very quick death after lung trapping, due to monocyte phagocytosis.^45^ The biodistribution of WJ‐MSCs has been explored after ^99m^Tc staining in a rat model of intracerebral haemorrhage. Whole‐body imaging carried out 2 hours after intravenous injection of WJ‐MSCs indicated activity in the lungs, liver, spleen, and kidneys. Gamma‐well counting at 24 hours after WJ‐MSC injection indicated higher uptake in the lungs, kidneys, spleen and liver, and very low activity in the brain.^58^ WJ‐MSCs may quickly disappear after intravenous infusion, at least in part because of their phagocytosis. In the mouse study by de Witte et al, intravenously injected WJ‐MSCs were rapidly phagocytosed by monocytes, which subsequently migrated from the lungs to other sites in the body. The phagocytosis of WJ‐MSCs‐induced phenotypic and functional changes in monocytes, which subsequently modulated cells of the adaptive immune system. The authors concluded that, at least in mice, monocytes play a crucial role in mediating, distributing and transferring the immunomodulatory effects of WJ‐MSCs.^45^ Still, WJ‐MSC tracking in humans after intravenous injection is unexplored. Recently, Galleu et al. demonstrated that BM‐MSCs rapidly underwent perforin‐dependent apoptosis by recipient cytotoxic cells after IV injection in vivo.^59^ After infusion, recipient phagocytes engulfed these apoptotic BM‐MSCs and produced IDO, which was ultimately necessary for effecting immunosuppression. Further studies with WJ‐MSCs are necessary to know if they are similarly subject to cytotoxicity and phagocytosis in humans.

Considering the fact that WJ‐MSCs probably disappear shortly after their injection and that some of their properties are linked to paracrine factors, it seems logical to compare their therapeutic properties with a cell‐free therapy, which could bypass some potential complications, for example viral infections, strong allo‐immunization or malignant transformation (even if this last event has not yet been described in vivo reports). Extracellular vesicles (EVs), which contain lipids, RNA, proteins and even DNA when derived from apoptotic cells, have been described as sharing the same properties as living WJ‐MSCs.^60^

In the field of haematopoiesis, EVs from BM‐MSCs and adipose‐derived MSCs have been reported to promote myeloid‐biased multipotent haematopoietic expansion in mice,^61^ and EVs from BM‐MSCs have been described as cell‐free biologics useful in the ex vivo expansion of HSCs,^62^ or as helping to rescue murine marrow haematopoietic cells from radiation damage.^63^ Although EVs derived from WJ‐MSCs have not been described in this area so far, the demonstration that WJ‐MSCs display a higher number of proteins related to haematopoietic cell differentiation, in comparison with other sources of MSCs,^25^ should lead to further specific research on the haematopoietic effects of EVs derived from WJ‐MSCs.

In the field of inflammation and GVHD, EVs from WJ‐MSCs have been reported as potent in vitro inhibitors of CD4^+^ T‐cell proliferation, through TGF‐β1 and adenosine signalling.^64^ In a mouse model of inflammatory bowel disease, EVs from WJ‐MSCs homed to colon tissues at 12 hours after injection and significantly relieved the severity of inflammatory bowel disease, with an effect similar to that of WJ‐MSCs used as controls, with a decrease of inflammatory cytokines in the colon and spleen and a modulation of IL‐7 expression in colonic macrophages.^65^ In a mouse model of GVHD, EVs from WJ‐MSCs prevented life‐threatening acute GVHD.^66^ Moreover, In patients with aGVHD, EVs having surface PDL‐1 have been identified after WJ‐MSC injections.^67^ However, three main points concerning the immune‐suppressive potential of WJ‐MSCs should be kept in mind: first, WJ‐MSCs depend on the inflammatory environment for their own secretion of immune‐suppressive soluble factors (described in Figure 1), since they may undergo polarization to either MSC1s or MSC2s according to the environment.^68^ EVs do not have this plasticity, and the priming of WJ‐MSCs with inflammatory cytokines should be considered before exosome collection in the field of GVHD.^69^ Second, WJ‐MSC phagocytosis by monocytes in vivo has been described as a key mechanism of immunomodulation,^45^, ^59^ and nothing is known about that phenomenon in regard to MSC‐derived EVs.

Third, cell preparation is an important variable that must be better defined before WJ‐MSC use in human therapy. For instance, since WJ‐MSCs are banked, the impact of freezing and thawing these cells before use must be carefully studied. Some authors have noted that BM‐MSCs need a few days of culture after thawing to recover their full immunological properties.^70^, ^71^, ^72^, ^73^ This was not confirmed in a preclinical pig model of septic shock where thawed human WJ‐MSCs were immediately infused and provided significant clinical and biological improvements.^74^ The culture medium^75^ and culture atmospheric conditions are also correlated with WJ‐MSC functions.^19^ Hypoxia has been described as a favourable condition in maintaining HSC stemness,^76^ but its impact on WJ‐MSC immune properties is not well characterized to date. The only published means of enhancing their immune capacities is to licence WJ‐MSCs with pro‐inflammatory cytokines ,^77^ for instance IFN‐γ, which improves their function in preventing GVHD in mouse models.^49^, ^78^ In the haematopoietic support setting as well, the cell preparation protocol appears to be of great importance to full WJ‐MSC potency. Three‐dimensional culture of MSCs with HSCs enhances the expansion of cord blood CD34^+^ cells.^79^ *Ex vivo* expansion of HSCs without eliminating the long‐term repopulating capacity of more primitive HSCs is more feasible when haematopoietic niches are mimicked.^80^ In this niche, cell contact between WJ‐MSCs and HSCs seems to be preferable.^34^ The surface structure of the microenvironment has also been shown to modify the cytokine secretion profile of MSCs.^81^ Even the stiffness of polydimethylsiloxane substrates for BM‐MSC culture can lead to a change in HSPC phenotype.^82^ Thus, the preparatory culture of MSCs in bioreactors might change their immunosuppressive and haematopoietic supportive properties. The addition of IL‐1β in the culture medium would also increase the haematopoietic support capacity of WJ‐MSCs.^83^ Finally, the route of administration of MSCs (intravenous or intrabone) could also change the ability of MSCs to support haematopoiesis.^84^

The next step to clinical translation is to produce a cellular therapy having reproducible immunosuppressive properties. The high inter‐individual variability observed in adult BM‐MSCs^85^ has been erased by pooling MSCs from eight voluntary donors.^14^ One idea would be to pool WJ‐MSCs from different cords to try to equalize their properties and decrease the variability of each therapeutic batch. However, the question of immunization against HLA expressed by allogeneic MSCs, especially in the case of MSCs pooled from several donors, is currently unresolved.

## 6 CONCLUSION

This is an updated review focused on WJ‐MSCs in haematology. It summarizes their immunosuppressive properties in vitro and in preclinical models and highlights the promise of WJ‐MSCs in haematology. They are a good candidate for haematopoietic support after cord blood transplantation, after alternative HSCT for aplastic anaemia, and for GVHD prophylaxis and treatment. To improve their potential, in‐vivo studies are still warranted in order to understand their biological distribution after intravenous injection in humans and their plasticity according to their preparation, and to decrease their inter‐individual variability in order to enhance the reproducibility of results. For this purpose, many ongoing clinical trials are seeking the best efficacy of WJ‐MSCs or their EVs in the field of allogeneic stem cell transplantation (for example, NCT01092026 in Belgium, NCT02032446 in Italy, NCT03847844 in Malaysia and NCT04738981 and NCT04213248 in China).

## CONFLICT OF INTEREST

The authors declare that they have no competing interests.

## AUTHOR CONTRIBUTIONS

**Cécile POCHON:** Writing – original draft (equal); Writing – review & editing (equal). **Anne‐Béatrice Notarantonio:** Writing – original draft (equal). **Caroline Laroye:** Writing – original draft (equal). **Loic Reppel:** Writing – original draft (equal). **Daniele Bensoussan:** Writing – original draft (equal); Writing – review & editing (equal). **Allan Bertrand:** Writing – review & editing (equal). **Marie‐Thérèse Rubio:** Supervision (equal). **Maud D'Aveni:** Supervision (equal); Writing – original draft (equal); Writing – review & editing (equal).

## Data Availability

Not applicable.
